# Functional Analysis of the Leading Malaria Vaccine Candidate AMA-1 Reveals an Essential Role for the Cytoplasmic Domain in the Invasion Process

**DOI:** 10.1371/journal.ppat.1000322

**Published:** 2009-03-06

**Authors:** Moritz Treeck, Sonja Zacherl, Susann Herrmann, Ana Cabrera, Maya Kono, Nicole S. Struck, Klemens Engelberg, Silvia Haase, Friedrich Frischknecht, Kota Miura, Tobias Spielmann, Tim W. Gilberger

**Affiliations:** 1 Department of Molecular Parasitology, Bernhard-Nocht-Institute for Tropical Medicine, Hamburg, Germany; 2 Hygiene Institut, Abteilung Parasitologie, Universitätsklinikum Heidelberg, Heidelberg, Germany; 3 Centre for Molecular and Cellular Imaging, EMBL, Heidelberg, Germany; National Institute for Medical Research, United Kingdom

## Abstract

A key process in the lifecycle of the malaria parasite *Plasmodium falciparum* is the fast invasion of human erythrocytes. Entry into the host cell requires the apical membrane antigen 1 (AMA-1), a type I transmembrane protein located in the micronemes of the merozoite. Although AMA-1 is evolving into the leading blood-stage malaria vaccine candidate, its precise role in invasion is still unclear. We investigate AMA-1 function using live video microscopy in the absence and presence of an AMA-1 inhibitory peptide. This data reveals a crucial function of AMA-1 during the primary contact period upstream of the entry process at around the time of moving junction formation. We generate a *Plasmodium falciparum* cell line that expresses a functional GFP-tagged AMA-1. This allows the visualization of the dynamics of AMA-1 in live parasites. We functionally validate the ectopically expressed AMA-1 by establishing a complementation assay based on strain-specific inhibition. This method provides the basis for the functional analysis of essential genes that are refractory to any genetic manipulation. Using the complementation assay, we show that the cytoplasmic domain of AMA-1 is not required for correct trafficking and surface translocation but is essential for AMA-1 function. Although this function can be mimicked by the highly conserved cytoplasmic domains of *P. vivax* and *P. berghei*, the exchange with the heterologous domain of the microneme protein EBA-175 or the rhoptry protein Rh2b leads to a loss of function. We identify several residues in the cytoplasmic tail that are essential for AMA-1 function. We validate this data using additional transgenic parasite lines expressing AMA-1 mutants with TY1 epitopes. We show that the cytoplasmic domain of AMA-1 is phosphorylated. Mutational analysis suggests an important role for the phosphorylation in the invasion process, which might translate into novel therapeutic strategies.

## Author Summary

Malaria is one of the most lethal parasitic diseases worldwide, causing more than 1 million fatalities per annum. Drug resistance is widespread, and a vaccine is not available. One of the leading blood stage vaccine candidates is the apical membrane antigen 1 (AMA-1), which is well-conserved among apicomplexan parasites. Although this protein plays an essential role in the invasion of human red blood cells, little is known about the molecular mechanisms underlying its function. In this study we use live video microscopy in the presence and absence of an invasion-inhibiting molecule to investigate the function of AMA-1 in real time. We establish a complementation assay based on strain-specific AMA-1 inhibition that allows functional characterization of this protein on a molecular level. Using this approach, we provide evidence that the intracellular cytoplasmic domain of AMA-1 is directly involved in the invasion process and that this domain is phosphorylated. We conclude that the phosphorylation of AMA-1 might represent an attractive target for novel therapeutic drug strategies independent of the polymorphic extracellular domain.

## Introduction

Invasion of red blood cells (RBCs) is one of the critical points in the erythrocytic life cycle of the malaria parasite *Plasmodium falciparum*. The invasive form of the parasite, the merozoite, harbours a set of specialized secretory organelles, in particular two varieties called rhoptries and micronemes, which house key proteins involved in the invasion process. Upon invasion, these proteins are released either onto the surface of the invading merozoite or into the intercellular matrix, where they mediate host-cell recognition, receptor binding, active invasion and the formation of the parasitophorous vacuole. Some of these proteins, like the apical membrane antigen-1 (AMA-1, PF11_0344), are primary targets for vaccine development since antibodies directed against these proteins can prevent invasion (reviewed in [1]). In *P. falciparum*, the full length AMA-1 protein is a 83 kD type I transmembrane protein stored in the micronemes [2],[3]. Upon egress of the merozoite from an infected erythrocyte, AMA-1 is translocated onto the merozoite surface where it is concentrated at the apical pole [4]. This translocation process is accompanied by N-terminal cleavage of a prodomain, which results in a 66 kDa protein that is itself further cleaved during invasion, releasing a 48 and a 44 kDa isoform into the extracellular environment [5],[6].

AMA-1 plays an essential role in the invasion process [7],[8] and is well conserved between apicomplexan parasites. However, its biological function is still unknown. Several studies have implicated *Plasmodium* AMA-1 function in erythrocyte binding [9],[10] and in reorientation of merozoites on the surface of RBCs [11]. More insights were gained in the related apicomplexan parasite *Toxoplasma gondii*, where it was shown that AMA-1 is involved in the regulation of rhoptry secretion and in mediating the formation of the moving junction, an area of intimate contact between the invading parasite and the host cell membrane [12]–[14]. However, precisely how AMA-1 mediates these multiple tasks in the extremely rapid invasion process remains a mystery.

There is significant polymorphism among *ama-1* alleles in *P. falciparum* field isolates [15],[16]. This diversity represents a major hurdle for the development of an AMA-1-based vaccine, as human driven immune selection leads to diversification of *ama-1* alleles [16],[17]. Significantly, it is known that invasion-inhibitory antibodies against the AMA-1 type of one parasite strain have no or significantly less efficacy against other parasite strains [17]. Residues responsible for this antigenic escape mechanism have been mapped [18] and co-crystallization studies reveal a hydrophobic cleft in the AMA-1 ectodomain as one of the binding sites for inhibitory antibodies [19]. This functional inactivation of AMA-1 can be mimicked by small peptides [20].

Detailed functional analysis of essential proteins like AMA-1 is hampered in *P. falciparum* by the limited availability of reverse genetic tools like RNAi or inducible gene knock-out systems [21]. Here we have generated a GFP-tagged full-length AMA-1 cell line that allows visualization of the surface translocation and assessment of the membrane mobility of this key protein in live parasites. Furthermore, we have established a complementation assay based on strain-specific inhibition to functionally characterize the crucial sequence determinants of AMA-1.

## Results

### Full-length AMA-1-GFP is correctly trafficked in transgenic parasites and is recognized by isoform-specific antibodies

**Figure 1:**
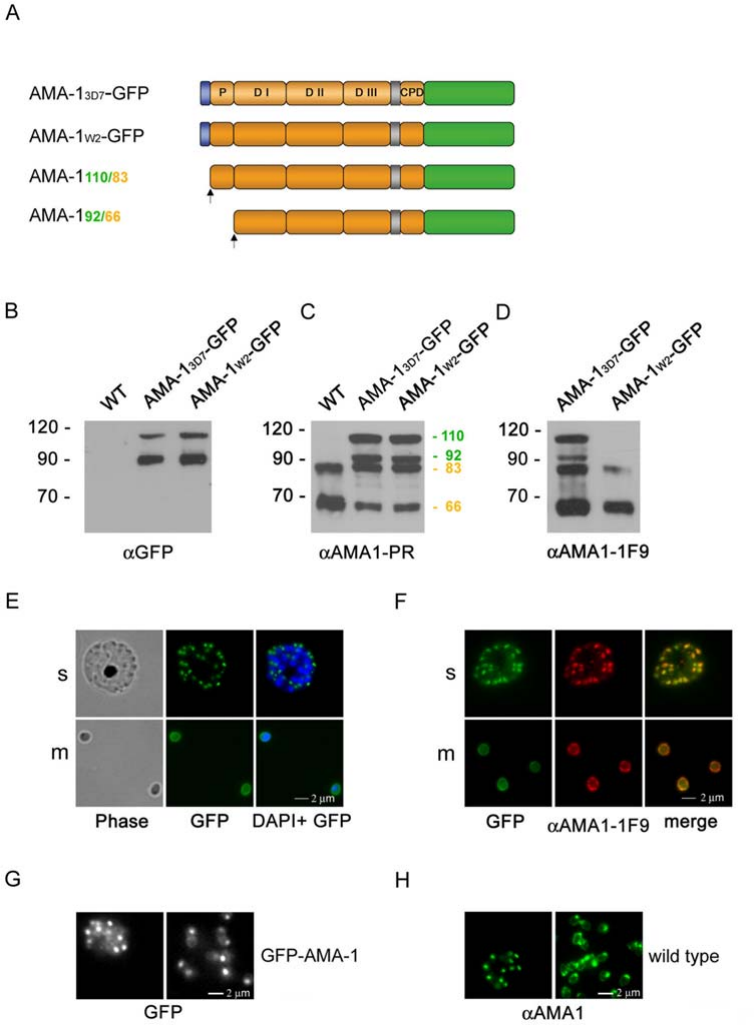
Expression and localization of 3D7- or W2mef-derived AMA-1 in transgenic parasites. (A) Schematic representation of episomally expressed AMA-1 GFP fusion proteins. Signal peptide: blue; transmembrane domain: grey; pro-domain (P); domain I-III (DI-DIII) and cytoplasmic domain (CPD): brown; GFP: green. 3D7-derived AMA-1 is represented in lighter brown colour. Cleavage sites of the signal peptide and the pro-domain are marked with arrows. Sizes of the processed proteins are indicated either in green (GFP fusion) or in brown (endogenous protein). (B) Western blot analysis of transgenic parasites using anti-GFP antibodies recognize 3D7- and W2mef-derived AMA-1-GFP proteins (the 110 kDa and the processed 92 kDa AMA-1-GFP fusion protein) in the transgenic parasite lines but not in wild type (WT) parasites. (C) Western blot analysis of transgenic AMA-1_3D7_-GFP and AMA-1_W2mef_-GFP expression using an AMA-1 specific polyclonal antibody. In wild type parasites, AMA-1 is recognized as an 83 kDa and a 66 kDa fragment (with and without pro peptide). In the transgenic cell lines, two additional proteins are apparent, representing the 110 kDa and the processed 92 kDa AMA-1-GFP fusion. (D) The monoclonal 1F9 antibody recognizes only the 3D7-derived but not the W2mef-derived AMA-1-GFP fusion protein. (E,F) Full-length AMA-1-GFP (green) localizes in schizonts (s) at the apical end of merozoites (m) and is distributed over the surface of merozoites after schizont rupture. This is shown in both unfixed (E) and fixed parasites (F). The distribution of the endogenous protein visualized with the 3D7-specific 1F9 antibody (red) is identical to the localization of the fusion protein (merge). (G,H) AMA-1-GFP dynamics during schizont rupture. (G) Time lapse microscopy of live AMA1-GFP–expressing parasites before and after schizont rupture. Strong apical GFP fluorescence with some surface staining was observed during schizont rupture and immediately after merozoite release (time frames from Video S1). (H) This distribution is indistinguishable from AMA-1 in wild type parasites visualized by a polyclonal AMA1 antibody (green).

It has been previously shown that AMA-1 is a microneme protein that is processed and translocated onto the surface of merozoites around the time of schizont rupture [6]. In order to functionally analyze AMA-1, full length AMA-1-GFP chimeras derived from either a 3D7 or W2mef background were episomally expressed under the AMA-1 promoter [22] in 3D7 parasites resulting in two parasite strains: AMA-1_3D7_-GFP and AMA-1_W2_-GFP (Figure 1A). Both chimeras are expressed and correctly processed as shown by Western blot analysis (Figure 1B and 1C). The expression and processing of endogenous AMA-1 is not affected by the ectopic expression of the transgenes (Figure 1C). The 3D7 specific, monoclonal antibody 1F9 exclusively recognizes AMA-1_3D7_-GFP but not AMA-1_W2_-GFP as shown in Figure 1D [23]. In order to confirm correct localization of the AMA-1-GFP fusion, we localized the protein in unfixed parasites (Figure 1E) and colocalized AMA-1_W2_-GFP with the endogenous protein in fixed parasites using the 3D7 specific monoclonal antibody (Figure 1F). The distribution of AMA-1-GFP is identical to endogenous AMA-1. The GFP-fusion protein is localized at the apical end of forming merozoites (s) and is distributed onto the surface in free merozoites (m). To visualize AMA-1 dynamics during schizont rupture and merozoite release, video fluorescence microscopy was undertaken using the AMA-1-GFP parasite lines (Figure 1G and Video S1 and Figure S1). Strong apical GFP fluorescence with some minor peripheral merozoite surface staining was observed prior to schizont rupture (Figure 1G, left) and immediately after merozoite release (Figure 1G, right). This AMA-1-GFP distribution is indistinguishable from AMA-1 in wild type parasites (Figure 1H) and in agreement with previously published studies of AMA-1 localization [24]–[26]. Over time, the initially apical AMA-1 becomes equally distributed over the periphery in free merozoites as shown in Figure 1E and 1F. To further analyze the mobility of peripheral AMA-1-GFP we used fluorescence recovery after photobleaching (FRAP) analysis of merozoites with predominantly peripheral GFP distribution (Figure S2). The qualitative analysis of the FRAP data revealed a high mobile fraction (89%+/−4%) which shows that most AMA-1 is not associated with an immobile entity and is freely diffusing (Figure S2) as it was previously suggested [26].

### Inhibition and complementation of endogenous AMA-1 using AMA-1-GFP–expressing parasites

**Figure 2:**
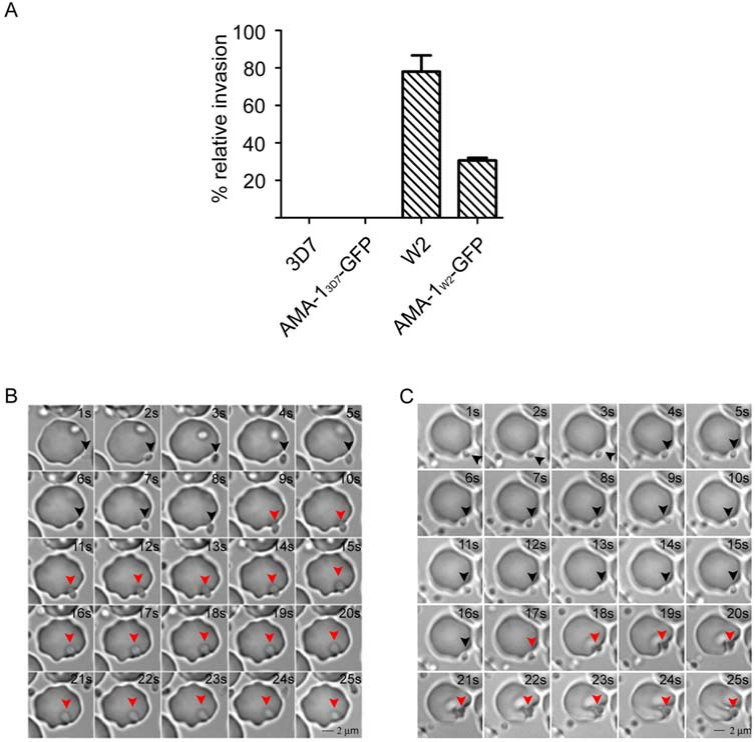
Inhibition and complementation of endogenous AMA-1 during invasion. (A) Invasion inhibition assays were performed in the presence of 100 µg/ml R1 peptide. The figure shows the percentage of invasion (relative invasion) compared to invasion in the absence of the inhibitory peptide. Error bars correspond to the standard deviation. All experiments were performed in triplicate in two independent experiments. In contrast to W2mef wild type parasites, 3D7 parasites cannot invade erythrocytes in the presence of R1 peptide. While over-expression of 3D7-derived AMA-1-GFP (AMA-1_3D7_-GFP) cannot restore the invasion capability of 3D7 parasites, the expression of W2mef-derived AMA-1 (AMA-1_W2mef_-GFP) in 3D7 parasites does. (B,C) Time lapse of video microscopy showing erythrocyte invasion in the absence or presence of R1 peptide. (B) A merozoite (arrowhead) attaches to the RBC, slowly reorients, and finally invades the erythrocyte. Reorientation is completed within 8 seconds, and the active invasion (red arrowheads) is completed after 12–16 seconds. Frame rate is 1 second, increment 1. (C) Time lapse of merozoites trying to invade a RBC in the presence of 100 µg/ml R1 peptide. Although AMA1 is functionally inactivated, the merozoite reorients (black arrowhead), and a forward movement can be observed but no invasion occurs (red arrowhead). Timeframe 1 s, increment 1.

To test the functional capacity of the episomally expressed AMA-1-GFP fusion protein, the endogenous AMA-1 of 3D7 parasites was functionally inactivated using a strain specific inhibitory peptide called R1 [20]. This peptide appears to bind to a hydrophobic pocket within the AMA-1 ectodomain [20]. In the presence of this peptide, invasion of 3D7 but not W2mef can be completely inhibited (Figure 2A). Ectopic expression of AMA-1_W2_-GFP in 3D7 parasites restored up to 50% of the invasion capability compared to W2mef wild type parasites, while AMA-1_3D7_-GFP did not compensate the inhibitory effect of the peptide (Figure 2A).

### Live video microscopy reveals a role for AMA-1 after the reorientation step

To reveal the consequences of functional inactivation of AMA-1 during invasion, parasites were observed by live video microscopy (Figure 2B and 2C and Videos S2, S3, S4, S5, S6, and S7, and Figures S3, S4, S5, and S6). As previously shown in *P. knowlesi* and *P. falciparum* [27]–[29] merozoites attach, reorientate (Figure 2B, black arrowheads) and subsequently invade the RBC (Figure 2B, red arrowheads, and Videos S2 and S3 and Figure S3). The primary attachment to the RBC surface is accompanied by oscillatory deformation of the erythrocyte surface [11],[27],[29] causing some extensive membrane- wrapping around the merozoite. After this attachment phase, the merozoite reorientates without apparent deformation of the RBC and invades the host cell within seconds (Videos S2 and S3 and Figure S3). After invasion, the infected erythrocyte goes through a reversible echinocytosis (Video S4 and Figure S4). In the presence of the R1 peptide, the invasion process by 3D7 or AMA-1_3D7_-GFP parasites appears to proceed normally through primary attachment and reorientation, but then fails to progress further (Figure 2C and Videos S5 and S6 and Figure S5). Interestingly, although host cell entry is blocked at this stage, echinocytosis still occurs (Video S7 and Figure S6), indicating that some decisive interactions with the RBC plasma membrane are not prevented in the presence of the R1 inhibitory peptide. This is further illustrated by a sudden forward movement which appears to exert a substantial force on the erythrocyte surface following reorientation (Videos S5, S6, and S7).

### Trafficking and translocation of AMA-1-GFP is independent of the cytoplasmic domain

**Figure 3:**
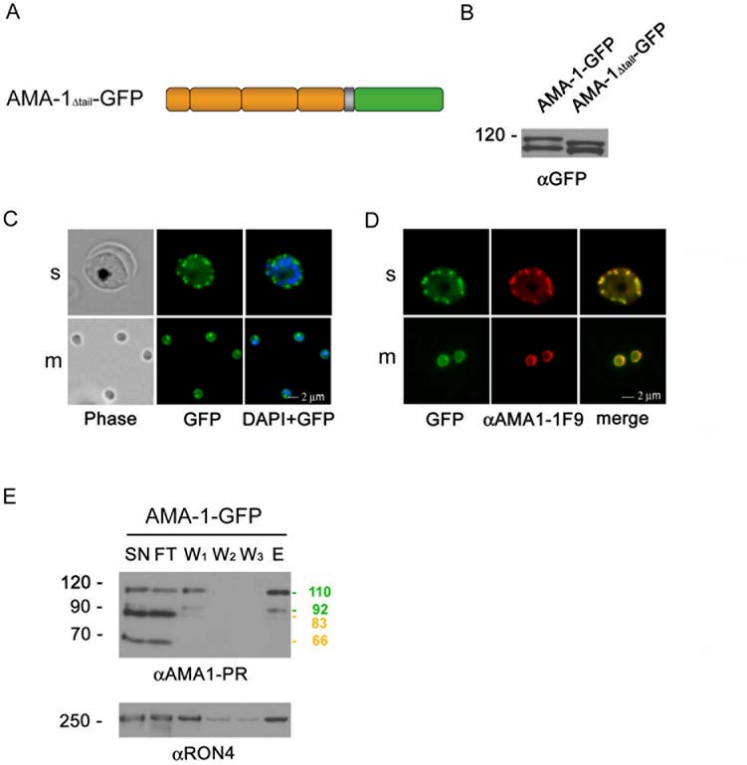
Expression and localization of mutant AMA-1-GFP. (A) Schematic representation of episomally expressed mutant AMA-1-GFP fusion proteins without cytoplasmic domain (AMA-1_Δtail_-GFP). (B) Expression of mutant AMA-1-GFP was verified using anti-GFP specific antibodies. (C,D) The deletion of the cytoplasmic domain does not affect the localization of the AMA-1_Δtail_-GFP fusion protein. This is shown in unfixed (C) and fixed (D) parasites. Mutant protein (green) colocalizes with the endogenous protein (red) as shown in the merge pictures. (E) Immunoprecipitation and immunoblot of AMA-1-GFP– interacting proteins. Coloured marker lines show the predicted size of the endogenous (brown), or the GFP-tagged AMA-1 (green). Supernatant (SN) of extract of AMA-1-GFP–expressing segmented schizonts was used on an AMA-1-GFP column. Supernatant (SN), flow through (FT), washing (W1-3), and the elution fraction (E) were separated on an SDS-PAGE gel and probed with polyclonal anti-PfAMA1 (PR) and anti-RON4.

In order to analyze the involvement of the AMA-1 cytoplasmic domain in microneme trafficking and subsequent surface translocation, W2mef-derived AMA-1-GFP–expressing 3D7 parasites lacking the cytoplasmic domain were generated (AMA-1_Δtail_-GFP, Figure 3A). Expression of mutant AMA-1-GFP was verified in immunoblots (Figure 3B). Fluorescence microscopy of unfixed (Figure 3C) or fixed (Figure 3D) transgenic parasites reveals that the deletion of the cytoplasmic domain does not affect AMA-1_Δtail_-GFP localization. A putative interaction (and therefore assisted trafficking) with the endogenous AMA-1 was excluded by using immunoprecipitation and subsequent Western blot analysis (Figure 3E). AMA-1-GFP and AMA-1_Δtail_-GFP (data not shown) were immunoprecipitated using anti-GFP beads. Although anti-AMA-1 antibodies showed successful co-immunoprecipitation of AMA-1-GFP and the AMA-1 binding partner RON4 [12], no endogenous AMA-1 could be detected. This argues against a piggy-back trafficking of the deletion mutant with the endogenous AMA-1.

### The AMA-1 cytoplasmic domain is essential for its function

**Figure 4:**
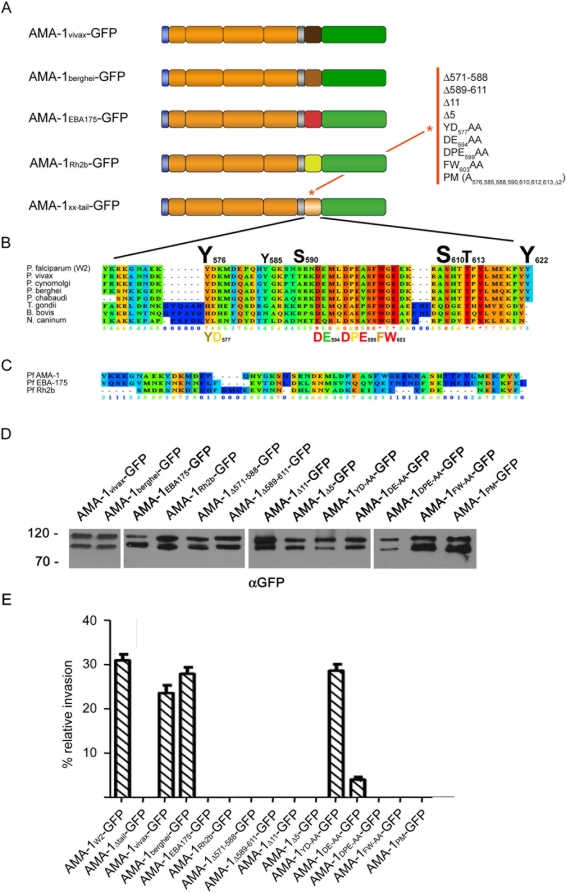
Functional analysis of the cytoplasmic domain of AMA-1. (A) Schematic representations of the mutations introduced into the cytoplasmic domain of W2mef-derived AMA-1. (B) Alignment of the cytoplasmic domain of various AMA-1 homologues. The conservation is scored and colour-coded by PRALINE (www.ibi.vu.nl). The scoring scheme works from 0 (for the least-conserved alignment position) up to 10 (for the most conserved position). Amino acids predicted by NetPhos (www.cbs.dtu.dk/services/NetPhos) to be phosphorylated in the plasmodial protein are scaled up according to their relative predicted probabilities. (C) Alignment of the cytoplasmic domain of AMA-1 with the cytoplasmic domain of EBA-175 and Rh2b. (D) Expression of the cytoplasmic domain mutants was verified by Western blot analysis using anti-GFP antibodies. (E) Invasion inhibition assays with all cytoplasmic domain mutants were performed in the presence of 100 µg/ml R1 peptide. Error bars correspond to standard deviation. All experiments were performed in triplicate in two independent experiments. The parasite line AMA-1_W2mef_-GFP served as a positive control. Except for the mutation of a conserved tyrosine (YD), all introduced mutations lead to a complete functional inactivation (in the case of the mutant DE, to a drastically reduced function) of AMA-1-GFP.

The complementation assay provided the basis for analyzing the phenotypic effects of various deletions and mutations introduced into the AMA-1 protein and as such to functionally characterize different parts of the AMA-1 molecule. First, we analyzed the effect of the cytoplasmic domain deletion. In the presence of the R1 peptide, W2mef-derived AMA-1_Δtail_-GFP could not rescue the invasion capability, unlike the full-length W2mef AMA-1-GFP (Figure 4E). Thus, although trafficking of AMA-1 does not require the cytoplasmic domain, invasion inhibition by R1 clearly demonstrates that the cytoplasmic domain (AMA-1_Δtail_-GFP) plays an essential role in the invasion process. To further dissect the role of the cytoplasmic domain we introduced multiple alterations into this domain and analyzed their functional consequences.

### Functional conservation within the cytoplasmic domain of AMA-1

In order to analyze the functional sequence requirements within the cytoplasmic domain of *P. falciparum* AMA-1, we swapped domains either with i) the homologous domain of *P. vivax* or *P. berghei* or ii) the non-homologous domains of two well characterized type-I transmembrane proteins, that are implicated in erythrocyte invasion and possess an equally short cytoplasmic domain (EBA-175 and Rh2b, Figure 4A–4C). All chimeric proteins were correctly expressed as GFP fusion proteins (Figure 4D), colocalized with endogenous AMA-1 (data not shown) and were proteolytically cleaved like the endogenous protein (Figure 4D). Although AMA-1_vivax_ and AMA-1_berghei_ could readily complement the endogenous protein, the cytoplasmic domains of EBA-175 and Rh2b failed to do so (AMA-1_EBA-175_ and AMA-1_Rh2b_, Figure 4E). This points towards distinctive features within the AMA-1 cytoplasmic domain that seem to be required for its function. This notion is further supported by a high degree of conservation of this domain not only within the genus *Plasmodium* but also with other apicomplexan parasites (Figure 4B). Therefore, we tested which region of the cytoplasmic domain is important for AMA-1 function. Deletions of the proximal (AMA-1_Δ571–588_ and AMA-1_Δ589–611_) and C-terminal (AMA-1_Δ11_ and AMA-1_Δ5_) portions of the cytoplasmic domain functionally inactivated AMA-1. Furthermore, mutations of the highly conserved residues DE_594_ (AMA-1_DE-AA_), DPE_599_ (AMA-1_DPE-AA_), FW_603_ (AMA-1_FW-AA_) all resulted in functional inactivation of AMA-1 with some residual function in the AMA-1_DE-AA_ parasite line. The only mutation that had no functional effect was YD_577_ (AMA-1_YD-AA_), a residue lying within the N-terminal part of the domain.

**Figure 5:**
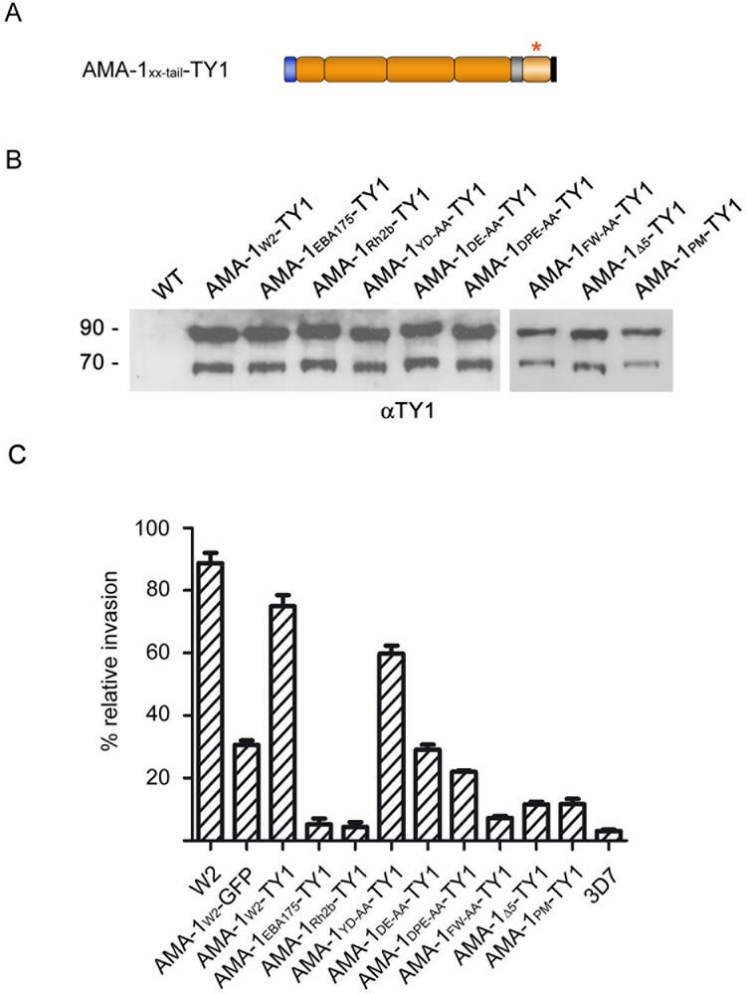
Expression and functional analysis of AMA-1-TY in transgenic parasites. (A) Schematic representations of the ectopically expressed TY1-tagged AMA-1 with various mutations in the cytoplasmic domain (lighter brown and marked with an asterisk). (B) Expression of W2mef-derived AMA-1-TY1 detected by Western blot analysis using an anti-TY1 antibody. Whereas no protein can be detected in wild type parasites, a double band corresponding to AMA-1_83_-TY1 and processed AMA-1_66_-TY1 can be detected in AMA-1-TY1–expressing parasites. (C) Invasion inhibition assays using AMA-1-TY1–expressing parasite strains. Assays were performed in the presence of 100 µg/ml R1 peptide and were performed in triplicate on three independent occasions.

To further validate these findings and to improve the invasion capability of parasite lines ectopically expressing AMA-1, the C-terminal GFP tag was exchanged with a small TY1 epitope of 10 amino acids [30] in wild type AMA-1 (AMA-1_W2_-TY1) and in those AMA-1 mutations that led to its inactivation (Figure 5A and 5B). The substitution of GFP with the TY1 tag increased the efficiency of the functional complementation by approximately two fold (Figure 5C) and led to an invasion capability of the AMA-1_W2_-TY1 parasite line that was comparable with that of W2mef wild type parasites (Figure 5C). The lack of invasion capability was not significantly changed in parasite lines substituted with non-homologous cytoplasmic domains (AMA-1_EBA-175_-TY1 and AMA-1_Rh2b_-TY1), or mutations within the extreme C-terminus (AMA-1_Δ5_-TY1, AMA-1_FW-AA_-TY1 and AMA-1_PM_-TY1). Evaluation of the TY1-tagged parasite lines AMA-1_DE-AA_ and AMA-1_DPE-AA_ revealed an increased invasion capacity, which however did not reach that of wild type AMA-1_W2_-TY1. The lower invasion efficiency with the GFP tag might be due to sterical hindrances compared to the much smaller TY1 tag.

### The cytoplasmic domain is phosphorylated

**Figure 6:**
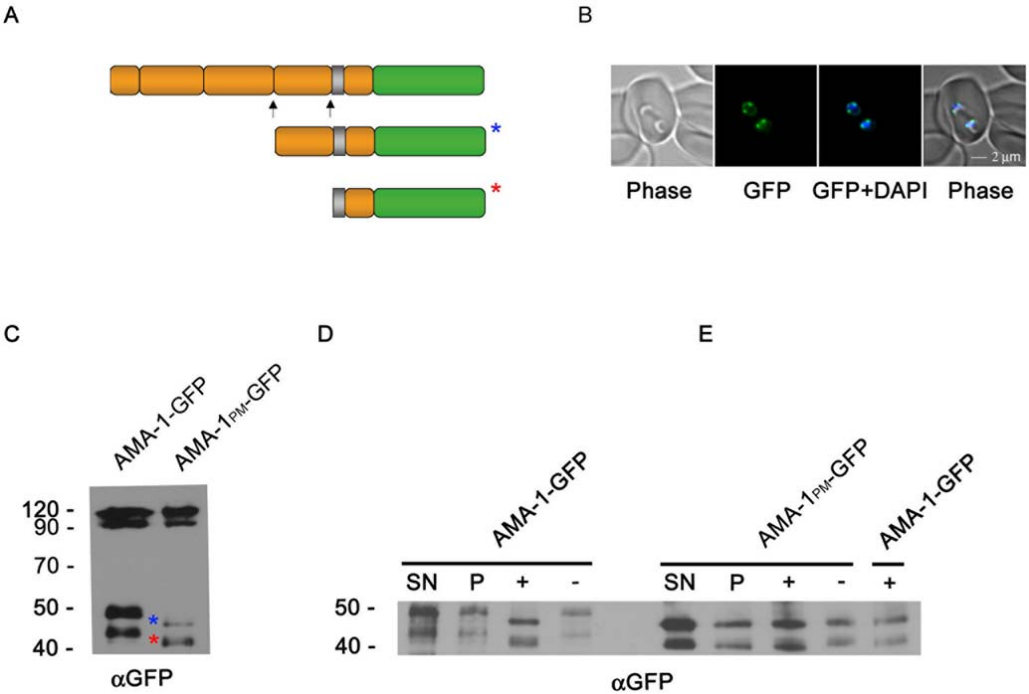
Phosphorylation of the cytoplasmic domain. (A) After invasion, secondary processing of AMA-1-GFP N- and C-terminal of domain III results in two additional AMA-1 GFP fragments of 44 and 49 kDa. Both fragments are indicated by coloured asterisks (49 kDa blue; 44 kDa red). (B) Live images showing AMA-1-GFP distribution (green) in two young ring stage or reinvaded merozoites. Blue: DAPI-stained nucleus. (C) Western blot analysis of AMA-1-GFP wild type and AMA-1-PM. Mutation of all putative phosphorylation sites to alanine within the cytoplasmic domain (PM, reducing the calculated MW by 0.6 kDa) leads to a reduced molecular weight of approximately 3 kDa of both fragments. (D) Western blot analysis of phosphatase-treated AMA-1-GFP. AMA-1-GFP was extracted and either treated with (+) or without (−) lambda-phosphatase followed by precipitation and then subjected to Western analysis. The pellet fraction (P) and the precipitated lysate supernatant (SN) without any treatment was loaded as a control. Treatment with lambda-phosphatase leads to a reduced molecular weight of approximately 3 kDa of both fragments, which is not seen in the control (−). (E) The treatment of the phosphorylation mutant (PM) with phosphatase does not affect the mobility of AMA-1-PM (with (+) and without (−) phosphatase treatment). Supernatant and pellet fraction served as controls. The size of all fragments is identical to that of the phosphatase-treated wild type AMA-1-GFP.

During the invasion process AMA-1 is shed by a protease from the merozoite surface releasing the extracellular domain into the supernatant (Figure 6A). The remaining small C-terminus of AMA-1 is carried into the host cell [24] (Figure 6B). A bioinformatics screen predicted six amino acids within the cytoplasmic domain to be phosphorylated (www.cbs.dtu.dk/services/NetPhos) (Figure 4B). To validate phosphorylation, all conserved putative phosphorylation sites (and those that might be phosphorylated due to the substitutions) were mutated to generate the parasite line AMA-1_PM_-GFP (Figure 4A). Taking advantage of the increased length of the processed C-terminal fragment due to the GFP tag, the released C-terminal fragments were easily detectable as 45 and 49 kDa fragments (Figure 6A and 6C). The introduced mutations (calculated to alter the MW by 0.6 kDa) led to a clearly reduced molecular weight (significantly more than 0.6 kDa) of both the 45 and the 49 kDa fragments (Figure 6C). In order to test if the observed shift in mobility was due to phosphorylation, AMA-1-GFP was treated with lambda-phosphatase (Figure 6D). This reduced the apparent molecular weight of wild type AMA-1-GFP, resulting in a size identical to that of the phosphorylation mutant AMA-1_PM_-GFP, whereas AMA-1_PM_-GFP itself showed no shift after the phosphatase treatment (Figure 6E). Thus the observed size difference between the mutated and wild type cytoplasmic domains of AMA1 is due to phosphorylation. Interestingly, this phosphorylation mutant (AMA-1_PM_) led to a functional inactivation of AMA-1 as shown in the complementation assays (Figures 4E and 5C).

## Discussion

The invasion of erythrocytes by malaria parasites is a well orchestrated and fast process relying on precise signaling and multiple protein-protein interactions [31]. Invasion can be resolved into a series of distinct steps: i) an initial attachment of the parasite and the host cell surface, which leads to a close interaction between the membranes of the two cells and often causes extensive deformation of the host cell, ii) reorientation of the merozoite, bringing about intimate contact between the apical end of the parasite and its host cell and iii) active penetration [27],[32],[33]. While some proteins involved in these steps have been characterized [31], the function of the leading blood stage vaccine candidate AMA-1 in this process remains elusive. Here we report on the detailed analysis of AMA-1 during invasion.

The expression of GFP-tagged full-length AMA-1 in transgenic parasites allowed us for the first time to visualize and analyze the distribution and function of AMA-1 during erythrocyte invasion. In agreement with previous publications [24]–[26], differentially distributed AMA-1 pools could be visualized: a major apically restricted population and a minor peripherally located fraction after merozoite egress. Does this imply dual function? On the one hand these different properties could argue for the involvement of peripheral AMA-1 in the reorientation process by its interaction with erythrocyte receptors [10],[11]. This could be achieved, for instance, by an AMA-1 density gradient with highest concentrations at the apical end. Apical AMA-1 could play an essential role in the intimate contact between the apical pole and host cell that culminates in the formation of a moving junction, a membrane contact that migrates down the length of the invading parasite. On the other hand, peripherally distributed AMA-1 could simply be functionally redundant excessive AMA-1, while only apical AMA-1 is required for invasion. Such a scenario was proposed for *T. gondii*, where it was shown that TgAMA-1 is associated with two proteins, RON2 and RON4, that colocalize with the moving junction [13]. Furthermore, the conditional knock-out of *Tgama-1* that resulted in its depletion impaired the ability of the parasite to attach intimately to its host cell [14]. These two observations argue against an essential role of AMA-1 in the steps preceding the formation of the moving junction, at least in *T. gondii*. Video microscopy in the presence of the invasion inhibitory peptide R1 was used to further examine AMA-1 function. To date only very few video recordings of the actual erythrocyte invasion process have been published [27]–[29] and none of these were made in the presence of invasion inhibiting molecules. The results obtained by comparing invasion in the presence and absence of R1 peptide suggest a role for the hydrophobic cleft just prior to invasion, after binding and reorientation (Figure 2 and Videos S3 and S5). Interestingly, the essential reorientation seems to be independent of erythrocyte deformation, strengthening the hypothesis of merozoite reorientation by either active reorientation with the use of motor proteins or a gradient of adhesive surface proteins with higher affinity or avidity towards the apical pole of the merozoite. The forward movement in the presence of the peptide indicates triggering of motor proteins even though invasion is inhibited. The apparent reorientation of the merozoite in the presence of the R1 peptide was surprising given that AMA-1 was shown to play an essential role in this process. This was previously shown by using inhibitory, monoclonal antibodies directed against *P. knowlesi* AMA-1 in combination with electron microscopy analysis [11]. One simple explanation for this differing observation could be that the *P. knowlesi* epitope recognized by inhibitory antibodies and the hydrophobic pocket in *P. falciparum* AMA-1 recognized by the R1 peptide are distinct functional domains of the AMA-1 ectodomain. This, once again, points to multiple functions of AMA-1 during the invasion process. Alternatively, the inhibition of the reorientation step by antibodies might be primarily mediated by a sterical hindrance due to the size of the antibodies and therefore might not reflect a functional property of the AMA-1 molecule.

The observed induction of echinocytosis in the presence of R1 peptide could argue for the initiation of a moving junction. Alternatively, transient echinocytes might be a consequence of transient lipid bilayer breaks caused by the force acting on the host cell during the invasion attempt, as reported for *T. gondii* [34]. Therefore, blocking of the hydrophobic pocket in the ectodomain of AMA-1 could either induce a blockage of the formation of the moving junction or the progression of the latter. One conceivable explanation for this observation is that R1 binding directly affects the interaction of AMA-1 with PfRON4 [12] or other unknown proteins involved in the moving junction complex. Alternatively, binding could interfere with conformational changes that might be necessary for AMA-1 function, potentially disrupting the essential role of the cytoplasmic domain. Functional knowledge about the cytoplasmic domains of invasion-related type I transmembrane proteins like AMA-1 is limited. Here we report that the cytoplasmic domain does not play a role in trafficking or translocation of the protein to the micronemes and subsequently to the surface, but is essential for function. This intriguing finding is reminiscent of previous work showing that although the cytoplasmic domain confers a function in the members of the EBL superfamily, these proteins are trafficked independently of their cytoplasmic domain [22],[35]. This is further supported by the fact that cytoplasmic domain-swaps with either EBA-175 or Rh2b do not interfere with trafficking but cannot complement AMA-1 function, pointing to a decisive role of this AMA-1 domain in the invasion process. This function can be mimicked by the homologous regions of the AMA-1 protein of *P. berghei* and *P. vivax* that show a high degree of conservation (above 70% identity within the cytoplasmic domains). In the cytoplasmic domain the degree of conservation increases towards the extreme C-terminus. Coincidentally, only mutations of residues positioned towards the transmembrane domain such as YD_577_ show no effect on AMA-1 function.

We show that the cytoplasmic domain of AMA-1 is phosphorylated and mutation of all putative phosphorylation residues inactivates AMA-1 function. Furthermore, the phosphorylation mutant, which possesses a fully functional AMA-1 ectodomain is blocked just prior to invasion, suggesting a coordinated function of the hydrophobic cleft and phosphorylation. In this context it is interesting to note that AMA-1 appears not to be phosphorylated prior to invasion but after schizont rupture: mass spectrometric peptide mass fingerprinting did not recover any phosphorylated AMA-1 in schizont material [5] (AMA-1_66_ fragment). It will be highly interesting to pinpoint the precise timing of AMA-1 phophorylation. This might help to unravel downstream effects triggered through AMA-1. Eight kinases are co-transcriptionally up-regulated together with AMA-1 [36]. Recently, Ono and co-workers [37] showed that the secretion of apical organelles is induced by the increase of the cAMP concentration in sporozoites of *P. falciparum* and other *Plasmodium* species. The major downstream effector of cAMP is protein kinase A (PKA), a serine threonine kinase l. Further, four of the up-regulated kinases display features of Ca^2+^-dependent kinases in *P. falciparum*. The dependence of efficient secretory organelle release on a raised concentration of intracellular Ca^2+^ is well established in *T. gondii* [38],[39], *Cryptosporidium parvum* [40] and was also suggested for *Plasmodium* [31]. Given that the cAMP and Ca^2+^ signaling pathways are intertwined in the parasite [41], the cytoplasmic domain of AMA-1 might function as a sensing device triggering downstream events like rhoptry secretion. It is noteworthy that the conditional AMA-1 knockout in *Toxoplasma* leads to defective rhoptry secretion [14]. Phosphorylation of the cytoplasmic domain might induce a conformational change in the extracellular domain resulting in its functional activation. Although phosphorylation seems to be a prerequisite for AMA-1 function, mutagenesis of conserved amino acids that are not predicted to be phosphorylated also interfered with AMA-1 function. These residues might be crucial for the interaction with downstream proteins that are involved in the invasion process. Alternatively, phosphorylation might depend on the recognition of surrounding non-phosphorylated residues. To further understand the functional effect of phosphorylation, it will be important to pinpoint the phosphorylation sites, the phosphorylating kinase(s), the downstream effector proteins, the precise moment of phosphorylation and then correlate this with individual invasion steps.

In summary, our findings provide evidence that AMA-1 is not only an essential part in the moving junction but also that phosphorylation of the cytoplasmic domain might be a prerequisite for invasion. This might translate into important novel therapeutic strategies using AMA-1-phosphorylating kinases as drug targets to inactivate this crucial player of erythrocyte invasion circumventing any antigenic escape mechanisms of the parasite.

## Materials and Methods

### Parasites strains and transfection

*Plasmodium falciparum* asexual stages were cultured in human 0^+^ erythrocytes according to standard procedures [42]. W2mef is derived from the Indochina III/CDC strain. 3D7 parasites were transfected as described previously [43] with 100 µg of purified plasmid DNA (Invitrogen). Positive selection for transfectants was achieved using 10 nM WR99210, an antifolate that selects for the presence of the human *dhfr* gene.

### Nucleic Acids and DNA constructs

*ama-1* was either amplified from 3D7 or W2mef *P. falciparum* cDNA (s. Tab. 1). Additionally, cDNA from *P. vivax* and *P. berghei* was used. *In vitro* mutagenesis of *ama1* was achieved by using a two-step primer directed PCR mutagenesis method [44] (Table S1) with proof reading Vent polymerase (NEB). To ensure correct timing of transcription, expression of the AMA-1 transgenes was controlled by the AMA-1 promotor using the pARL-AMA1-GFP Vector [22]. Constructs were cloned into the KpnI and AvrII restriction sites of the transfection vector and sequences were confirmed by sequencing.

### Antisera and immunoblots

Antibodies used in immunodetection were rabbit polyclonal anti AMA-1 [26] anti AMA-1 monoclonal 1F9 [23], monoclonal anti TY1 (Diagenode) and monoclonal anti-GFP (Roche). Anti-AMA-1 was diluted 1∶500, anti TY1 was used 1∶1500, 1F9 and anti-GFP antibodies were diluted 1∶1000 in phosphate-buffered saline (PBS) with 3% w/v skim milk. Immunoblots were performed using standard procedures and developed by chemiluminescence using ECL (Amersham International). Secondary antibodies were sheep anti-rabbit IgG horseradish peroxidase (Sigma) and sheep anti mouse IgG horseradish peroxidase (Roche) used 1∶5000.

### Immunofluorescence assays

Immunofluorescence assays (IFAs) were performed on fixed parasites as previously described [35],[45]. Fixed parasites were incubated for 1 h with primary antibodies in the following dilutions: rabbit anti-AMA-1 (1∶1000), mAB 1F9 (1∶2000). Subsequently, cells were incubated 1∶2000 with Alexa-Fluor 594 goat anti-rabbit IgG or Alexa-Fluor 488 goat anti-mouse IgG antibodies (Molecular Probes) and with DAPI at 1 µg/ml (Roche). Images of GFP-expressing parasites and immunofluorescence assays were observed and captured using a Zeiss Axioskop 2plus microscope, a Hamamatsu Digital camera (Model C4742-95) and OpenLab software version 4.0.4 (Improvision Inc.).

### Confocal and video microscopy

Confocal pictures were generated using a Fluoview 1000 (Olympus) or a SP5 (Leica) confocal microscope. Images were analyzed and processed using either Photoshop or ImageJ software (rsb.info.nih.gov/ij/). Parasites were taken from blood-culture, kept at 37°C and imaged immediately after withdrawal.

Video microscopy was performed using an Axiovert 200 M “Cell observer” with a temperature and CO_2_ controlled incubator at 37°C and 5% CO_2_. Briefly, parasites from culture where diluted 1∶100 in pre-warmed complete Media (Gibco) and 5% Albumax, (Sigma) without phenol red. 100 µl of this suspension was imaged either with, or without 100 µg/ml R1 inhibiting peptide in 35 mm glass bottom culture dishes (MatTek, Ashland, MA, USA). Image acquisition was performed with an AxioCam HRm camera (Zeiss) and Axiovision (Zeiss).

### Immunoprecipitation

AMA-1-GFP was immunoprecipitated using conjugated anti-GFP antibody agarose beads (MBL). Briefly, synchronized late parasite cultures were collected and saponin lysed. The parasite pellet was incubated with lysis-buffer (50 mM Tris-HCL pH 7,2, 250 mM NaCl, 0,1% NP-40, 2 mM EDTA, 10% Glycerol) containing complete protease inhibitor without EDTA (Roche) for 3 h at 4°C. After centrifugation for 30 min at 16.000 g the supernatant was incubated with anti-GFP agarose beads for 1 h at 4°C. Beads were washed three times with lysis buffer. Bound proteins were eluted with non-reducing SDS sample buffer and cooked for 5 min. Supernatant, flow through, wash and elution fractions were subjected to Western blot analysis. The Blot was probed using either polyclonal anti-AMA-1-PR or monoclonal anti-RON4 antibodies.

### Erythrocyte invasion assays

Parasite erythrocyte invasion assays were performed using 3D7 and transgenic parasites expressing various AMA-1 mutants in the 3D7 background. Parasitemia was measured using a Becton-Dickinson FACSaria fluorescence activated cell sorter (FACS). Sorbitol synchronized parasite culture [46] (4% hematocrit, 0,5- 1% parasitemia, late trophozoites) was incubated in a 96-well Plate (100 µl per well) under standard culturing conditions for 48 hours to allow reinfection in the presence or absence (control) of 100 µg/ml R1 [20]. After reinvasion occurred, parasites where stained with 1 mg/ml ethidium bromide for 30 minutes at 37°C, washed three times with media and then counted using the FACS. Assays were performed in triplicates on three independent occasions.

### Phosphatase assays

Sorbitol synchronized parasites were harvested after reinfection of erythrocytes. Parasites were released from host cells by saponin-lysis. The parasite pellet was resolved in 10 volumes of ice cold lysis buffer (50 mM Tris-HCL pH 7,2, 250 mM NaCl, 0,1% NP-40, 10% Glycerol) containing complete protease inhibitor (Roche) w/o EDTA and incubated for 180 minutes at 4°C. The lysate was centrifuged at 16.000 g and aliquots of the supernatant were subjected to lambda protein phosphatase (λPPase, NEB Biolabs) treatment as stated in the manufacturer's instructions. Briefly, 160 µl supernatant was incubated for 15 minutes with 400 U of λPPase at 30°C. Subsequently, the mixture was precipitated with 4 volumes of ice-cold acetone (Sigma). Precipitated protein was dissolved in non-reducing SDS sample buffer and analyzed on SDS-Page and Western blot analysis using anti-GFP antibodies.

## Supporting Information

Figure S1 Video S1 file depicted in time-lapse micrographs
(0.4 MB TIF)

Figure S2FRAP analysis of surface exposed AMA-1-GFP in free merozoites. (A) Representative pictures of a bleached merozoite at different time points PB (pre bleach), B (bleach pulse), post B (post bleach), and 2 s ABP (2 seconds after the bleach pulse). The bleached area is indicated as white area. FRAP data was collected in 13 independent experiments. (B) Kinetics of the fluorescence for every single region of interest (ROI). Positions of the ROIs are indicated in the inlay. The bleach impulse was at 0.6 seconds. All ROIs in the cell are affected by the bleach pulse. In the bleached area, fast recovery can be observed (green). In the apical ROI (violet) the loss of fluorescence is delayed by 0.2 seconds. 50% of surface fluorescence (violet) is lost within 0.157 seconds. Background fluorescence was measured in the rectangular ROI (orange). (C) Qualitative analysis: the FRAP curve was normalized by pre-bleach fluorescence intensity. Time point zero indicates the bleach impulse when recovery starts. Fluorescence recovery halftime (t/2) in the bleached area was 0,148 s (+/−0.04 s), and the mobile fraction was calculated to be 89% (+/−4%). Method: For analysis of the mobility of AMA-1-GFP on the merozoite surface, a small bleach area was chosen and bleached with a single pulse using Olympus Fluoview 1000. Due to the relatively small amount of GFP in a small area, the pinhole was set to 2 Airy. The data was analyzed using the IgorPro script K_FRAPcalcV9e.ipf by Kota Miura (http://www.embl.org/cmci/downloads/frap_analysis.html) in the software IgorPro (Wavemetrics) using the protocol proposed by Phair et al. (2004). FRAP curves were normalized using the following formula:
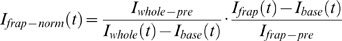
(1)Ifrap(t) is the temporal change in the intensity at the frap-ROI, Ibase(t) is the background intensity outside cells, Ifrap-pre is the average intensity before the bleaching at the frap-ROI, Iwhole(t) is the temporal changes in the intensity of the whole cell, Iwhole-pre is the average intensity of the cell before the bleaching. Then the normalized FRAP curve Ifrap-norm(t) was fitted with the following equation:
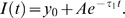
(2)Mobile fraction was calculated by
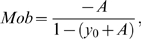
(3)and the half-maximum intensity time was calculated by
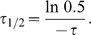
(4)Graphs where generated in Graphpad Prism5 or Igorpro.(0.6 MB TIF)

Figure S3 Video S3 file depicted in time-lapse micrographs
(0.3 MB TIF)

Figure S4 Video S4 file depicted in time-lapse micrographs
(0.4 MB TIF)

Figure S5 Video S6 file depicted in time-lapse micrographs
(0.3 MB TIF)

Figure S6 Video S7 file depicted in time-lapse micrographs
(0.5 MB TIF)

Table S1 Primers used for this study. Restriction sites for cloning are underlined.
(0.04 MB DOC)

Video S1 AMA-1 distribution during merozoite egress. AMA-1-GFP–expressing schizont shows GFP fluorescence concentrated in the apical tip of merozoites, consistent with a localization in the micronemes. Prior to schizont rupture, the merozoite movement becomes agitated before getting explosively released. Immediately after merozoite release, AMA-1-GFP is distributed predominantly in the apical area, but some fluorescence can be detected in the peripheral part of the merozoite. 1 frame per second, movie length: 303 seconds.
(1.8 MB MOV)

Video S2 Invasion of a merozoite into erythrocytes. A schizont is bursting, thereby releasing multiple merozoites into the culture media. A single merozoite can be observed to re-infect a new erythrocyte. 1 frame per second, movie length: 149 seconds.
(7.3 MB MOV)

Video S3 Section of Video S2. A section of Video S2 illustrates the different steps in the invasion process. The merozoite re-orientates on the surface without noticeable deformation of the erythrocyte. The invasion is a smooth and continual process. 1 frame per second, movie length: 24 seconds.
(0.4 MB MOV)

Video S4 Deformation and subsequent restoration of the erythrocyte during the invasion process. Merozoites attack an uninfected erythrocyte. At least one merozoite invades the erythrocyte after several contacts with accompanying re-orientation and deformation of the erythrocyte surface (10 s–44 s). Approximately 40 s after invasion, echinocyte morphology is induced that is recovered after another approximately 40 s. 1 frame per second, movie length: 294 seconds.
(14.4 MB MOV)

Video S5 Erythrocyte invasion is blocked in the presence of the R1 peptide. Initial binding and intimate contact with the erythrocyte membrane is not impacted by the inactivation of AMA-1 due to R1 peptide binding. 1 frame per second, movie length: 101 seconds.
(3.5 MB MOV)

Video S6 A section of Video S5 illustrates the different steps in the R1 blocked invasion process. Although the merozoite re-orientates on the surface as shown in Video S2 (without R1 peptide), the apical attachment does not culminate in the invasion of the erythrocyte. Noteworthy, after apical attachment and a short time period with no apparent movement, the exertion of strong force leads to local deformation of the erythrocyte membrane wrapping around the merozoite.
(0.7 MB MOV)

Video S7 Induction of echinocyte morphology in the presence of R1 peptide. Although the parasite is not able to proceed with the invasion process after its re-orientation on the RBC surface, it is able to induce very similar morphological changes observed in the invasion process in the absence of R1 peptide.
(7.1 MB MOV)
