# Supplementary material for: Functional Analysis of the Leading Malaria Vaccine Candidate AMA-1 Reveals an Essential Role for the Cytoplasmic Domain in the Invasion Process
Source: PLoS Pathog. 2009 Mar 6;5(3):e1000322. doi: 10.1371/journal.ppat.1000322 (PMC2654807; doi:10.1371/journal.ppat.1000322)
Supplement: Table S1 — Primers used for this study. Restriction sites for cloning are underlined. (0.04 MB DOC) [file ppat.1000322.s007.doc]

**Treeck et al., Tab. 1**

| **Primer** | Sequence |
| --- | --- |
| AMA1-S | GCGCGGTACCATGAGAAAATTATACTGCGT |
| AMA1-AS | GCGCCCTAGGATAGTATGGTTTTTCCATCAG |
| AMA1-Δ_tail_ | GCGCCCTAGGAGCATTTCCTTTTCTTTTATAAAGATAAACC |
| AMA1/RH2b-S | GCAACTATTTTAATGGTTTATCTTTATAGATCAAATAAGGATGAATGCGAT |
| RH2B-AS | GCGCCCTAGGAAAATATTTTTCTTCATTTTCATCAAACAATTTC |
| AMA1/EBA175-S | GCAACTATTTTAATGGTTTATCTTTATCAAGCCAAATATCAATCTGAAGGAGTTATG |
| EBA175-AS | GCGCCCTAGGTATCTTAAATTTAATATC ATAAGACTTTG |
| AMA1-W2/vivax-S | GCAACTATTTTAATGGTTTATCTTAGGAAGAAGGCTAACAATGATAAGTATG |
| AMA1-W2/vivax-AS | CATACTTATCATTGTTAGCCTTCTTCCTAAGATAAACCATTAAAATAGTTGC |
| AMA1-vivax-AS | GCGCCTCGAGGTAGTACGGCTTCTCCATCAGCACGG |
| AMA1-W2/berghei-S | GCAACTATTTTAATGGTTTATCTTAAAAGTAATAAAAAAGGTGAAAATTATG |
| AMA1-W2/berghei-AS | CATAATTTTCACCTTTTTTATTACTTTTAAGATAAACCATTAAAATAGTTGC |
| AMA1-berghei-AS | GCGCCTCGAGATAGTATGGTTTTTCCATCAGAACTG |
| AMA1-Δ_5_-AS | GCGCCCTAGGCATCAGAACTGGTGTTGTATGTG |
| AMA1-Δ_11_-AS | GCGCCCTAGGATGTGATGCTCTTTTTTCTTCCC |
| AMA1-DE_594_-AA-S | CAAGAAATGCTGCAATGTTAGATCC |
| AMA1-DE_594_-AA-AS | GGATCTAACATTGCAGCATTTCTTG |
| AMA1-FW_603_-AA-S | CCTGAGGCATCTGCTGCGGGGGAAG |
| AMA1-FW_603_-AA-AS | CTTCCCCCGCAGCAGATGCCTCAGG |
| AMA1-DPE_599_-AA-S | GCGGCGGCATCTTTTTGGGGGGAAG |
| AMA1-DPE_599_-AA-AS | CTTCCCCCCAAAAAGATGCCGCCGCTAACATTTCATCATTTCTTGAATTTG |
| AMA1-YD_576_-AA-S | GGAAATGCTGAAAAAGCTGCTAAAATGGATG |
| AMA1-YD_576_-AA-AS | CATCCATTTTAGCAGCTTTTTCAGCATTTCC |
| AMA1-Δ_571-588_-S  AMA1-570-AS | CTATTTTAATGGTTTATCTTTATAAAAGAAAAGCAGCTGCTAATTCAAGAAATGATGAAATGTTAGATCCTGAGGC  TTTTCTTTTATAAAGATAAACCATTAAAATAG |
| AMA1-Δ_589-611_-AS | GCGCCCTAGGATAGTATGGTTTTTCCATCAGAACTGGTGTTGTATGATTTCTTGAATTTGATTTCCCATA |
| AMA1-TY1- AS | GCGCCTCGAGTTAATCAAGTGGATCTTGATTTGTATGCACTTCCCTAGGATAGTATGGTTTTTCCATCAGAACTGGTGTTG |
